# Biocontrol Potential of Sodin 5, Type 1 Ribosome-Inactivating Protein from *Salsola soda* L. Seeds

**DOI:** 10.3390/biom14030336

**Published:** 2024-03-12

**Authors:** Monika Novak Babič, Sara Ragucci, Adrijana Leonardi, Miha Pavšič, Nicola Landi, Igor Križaj, Nina Gunde-Cimerman, Kristina Sepčić, Antimo Di Maro

**Affiliations:** 1 Department of Biology, Biotechnical Faculty, University of Ljubljana, 1000 Ljubljana, Slovenia; monika.novakbabic@bf.uni-lj.si (M.N.B.); nina.gunde-cimerman@bf.uni-lj.si (N.G.-C.); kristina.sepcic@bf.uni-lj.si (K.S.); 2 Department of Environmental, Biological and Pharmaceutical Sciences and Technologies (DiSTABiF), University of Campania ‘Luigi Vanvitelli’, 81100 Caserta, Italy; sara.ragucci@unicampania.it (S.R.); nicola.landi@unicampania.it (N.L.); 3 Department of Molecular and Biomedical Sciences, Jožef Stefan Institute, 1000 Ljubljana, Slovenia; adrijana.leonardi@ijs.si (A.L.);; 4 Department of Chemistry and Biochemistry, Faculty of Chemistry and Chemical Technology, University of Ljubljana, 1000 Ljubljana, Slovenia; miha.pavsic@fkkt.uni-lj.si; 5 Institute of Crystallography, National Research Council of Italy, 81100 Caserta, Italy

**Keywords:** agretti, antifungal activity, bioinsecticide, halophilic fungi, mass spectrometry, ribosome-inactivating protein

## Abstract

Sodin 5 is a type 1 ribosome-inactivating protein isolated from the seeds of *Salsola soda* L., an edible halophytic plant that is widespread in southern Europe, close to the coast. This plant, known as ‘agretti’, is under consideration as a new potential crop on saline soils. Considering a possible defence role of sodin 5 in the plant, we report here its antifungal activity against different halophilic and halotolerant fungi. Our results show that sodin 5 at a concentration of 40 µg/mL (1.4 µM) was able to inhibit the growth of the fungi *Trimmatostromma salinum* (35.3%), *Candida parapsilosis* (24.4%), *Rhodotorula mucilaginosa* (18.2%), *Aspergillus flavus* (12.2%), and *Aureobasidium melanogenum* (9.1%). The inhibition observed after 72 h was concentration-dependent. On the other hand, very slight growth inhibition was observed in the fungus *Hortaea werneckii* (4.2%), which commonly inhabits salterns. In addition, sodin 5 showed a cytotoxic effect on the Sf9 insect cell line, decreasing the survival of these cells to 63% at 1.0 µg/mL (34.5 nM). Structural analysis of sodin 5 revealed that its N-terminal amino acid residue is blocked. Using mass spectrometry, sodin 5 was identified as a homologous to type 1 polynucleotide:adenosine glycosylases, commonly known as ribosome-inactivating proteins from the Amaranthaceae family. Twenty-three percent of its primary structure was determined, including the catalytic site.

## 1. Introduction

The term ‘ribosome-inactivating proteins’ (RIPs) refers to plant proteins that inactivate ribosomes [1,2], although analogous toxic proteins have also been found in fungi and bacteria [3,4]. RIPs are often produced by plants in several isoforms [5,6]. Their physiological role is still controversial. It has been hypothesized that plants accumulate RIPs in some of their tissues for defence against biotic and abiotic stresses [7,8].

RIPs are N-β-glycosylases (EC 3.2.2.22) that remove a single adenine from the Sarcin-Ricin Loop (SRL) of conserved 28S rRNA [9]; thereafter, the ribosome is unable to bind the elongation factors, resulting in translation blockage [10]. In addition, various RIPs are able to depurinate stretches of adenosine repeats from herring sperm DNA, poly(A) and viral RNA, for which they are known as polynucleotide:adenosine glycosylases (PNAGs) [11,12].

Structurally, RIPs from plants are currently classified into three types based on their physical properties and the presence or absence of a lectin-like chain: type 1, type 2, and type 3. Type 1 RIPs are single-chain basic enzymes (pI ≥ 9) with an approximate molecular mass of 30 kDa [1]. Studies on protein and DNA sequences showed that most type 1 RIPs are synthesized as preproteins [13]. Type 2 RIPs typically have two chains and an approximate molecular mass of 60 kDa, although some oligomeric forms have also been found [6]. The chain with enzymatic activity is named A-chain, while the lectin-like chain is the B-chain. The latter is linked to the A-chain by disulphide bonds and other non-covalent attractive forces [1]. Type 2 RIPs are generally more cytotoxic than type 1 RIPs. The presence of the B-chain facilitates the translocation of the A chain into the cytosol by binding to galactosyl moieties of glycoproteins and/or glycolipids on the surface of eukaryotic cells [14]. Type 3 RIPs are synthesized as single-chain inactive precursors (pro-RIPs) that transform into two-chain type 1-like RIPs after proteolytic processing [15].

RIPs have attracted considerable interest in recent years due to their potential applications in agriculture and medicine [16]. The potential use of RIPs in agriculture is due to their antiviral, antifungal and insecticidal activities to improve plant defence [17,18], while in medicine RIPs are known for their cytotoxic activity against various malignant cells. Indeed, several IgG-tagged RIPs (the IgG makes the toxin selective for a specific target) have been tested for the treatment of various tumours [19]. In addition, other efficient carriers such as cell-binding ligands, protease inhibitors, hormones, and nanomaterials (inorganic nanoparticles, liposomes, and polymers) have been fused or chemically conjugated to different RIPs members in order to create specific bifunctional cytotoxic agents [16]. On the other hand, research on RIPs indicates a novel potential role for these translation inhibitors, as potential transgenes in developing transgenic plants to combat multiple stresses in the environment. Indeed, RIPs target the fundamental processes of the cell with very high specificity to the infecting pests, and at the same time cause the ectopic expression of pathogenesis-related proteins and trigger both enzyme-dependent and enzyme-independent metabolic pathways, alleviating multiple environmental stresses such as drought, salinity, and temperature [20].

Our group has recently isolated and thoroughly characterized several type 1 RIPs from the seeds, roots, and leaves of the barilla plant, or ‘agretti’ in the local language (*Salsola soda* L.) [21]. In particular, the major RIP isoform named sodin 5 (~29 kDa) was isolated from the seeds of *S. soda* with a yield of ~2.9 mg/100 g seeds. Sodin 5 inhibited protein synthesis in a cell-free system (IC_50_ = 4.83 pM). It showed a high thermal stability, and its melting temperature (Tm) was ~76 °C. Moreover, sodin 5 exhibited interesting biological activities, such as a cytotoxic effect against HeLa and COLO 320 cell lines, as well as apoptotic and antifungal activity against the necrotrophic fungus *Penicillium digitatum*, which is involved in the postharvest decay of citrus fruits [21].

The presence of sodin 5 and other type 1 RIPs in *S. soda* is of interest as it is an edible plant and a potential new crop [22]. *S. soda* is a halophyte which is frequently cultivated along the Mediterranean coasts and can be irrigated with saltwater [23]. Moreover, several authors have reported the possible use of this plant as an alternative crop to rehabilitate unproductive saline soils [22]. In addition, the use of *S. soda* as a desalinating companion plant has a beneficial effect on the growth, yield, mineral composition, and quality of salt-sensitive crops (i.e., *Lactuca sativa* L. (lettuce) [24], *Lycopersicon esculentum* Mill. (tomato) [25], and *Capsicum annuum* L. (greenhouse pepper) [26]).

In this context, and in view of the potential use of *S. soda* as an alternative crop on salty soils, we report here a partial primary structure of sodin 5 and its activity against certain halophilic fungi (*Aureobasidium melanogenum*, *Hortaea werneckii*, *Rhodotorula mucilaginosa,* and *Trimmatostromma salinum*) [27,28] and halotolerant fungi *Aspergillus flavus* and *Candida parapsilosis* [29]. Selected fungi are commonly associated with salty habitats [30,31]. Although some studies have reported an association between *S. soda* and a number of different plant- and soil-associated fungi [32,33], none of the species tested were directly isolated from *S. soda*. The results of the present study thus contribute to the knowledge of the synergistic and antagonistic effects of sodin 5 on fungi co-inhabiting salty habitats with *S. soda*. We also tested the effect of sodin 5 on yeasts, which are often isolated from anthropogenic habitats and therefore found in seawater and shores [34,35,36]. In addition, the effect of sodin 5 on the Sf9 (*Spodoptera frugiferda*) insect cell line was tested to verify its insecticidal potential [17].

Our findings lay the foundation for future analyses that would include fungal plant pathogens exposed to a wider concentration range of sodin 5. Determining the effect of sodin 5 on fungi and animal cell lines contributes to future biotechnological applications of the protein, where the effective dosages could replace standard fungicides.

## 2. Materials and Methods

### 2.1. Materials

The chemicals for chromatography were previously reported [21,37]. Single-stranded salmon sperm DNA was obtained from Sigma-Aldrich Solutions (Merk Life Science, Milan, Italy). The nuclease-treated rabbit reticulocyte lysate system was purchased from Promega (Madison, WI, USA). Other chemicals (analytical grade) were from Sigma-Aldrich Solutions. Insect cells derived from the ovarian epithelial cells of the fall army worm (*Spodoptera frugiperda*; Sf9 cells; Thermo Fisher Scientific, Waltham, MA, USA) were maintained in continuous suspension culture under serum-free conditions at 27 °C in ESF 921 protein-free insect cell medium with L-glutamine (Expression Systems, Davis, CA, USA), with agitation at 150 rpm.

### 2.2. Protein Purification

Sodin 5 was prepared as previously described by Landi et al. [21], from *S. soda* seeds using a general protocol for the preparation of basic type 1 RIPs [4,37,38]. Briefly, soluble basic proteins obtained after acid precipitation at pH 4.0 by using glacial acetic acid were fractionated by several sequential chromatographic steps: (i) cationic chromatography with stepwise elution (SP-Stream line resin; Cytiva Italia S.r.l., Buccinasco, MI, Italy); (ii) gel-filtration chromatography (HiLoad 26/60 Superdex 75 prep grade; Cytiva Italia S.r.l.); and (iii) cationic-exchange chromatography (S-Sepharose resin, Cytiva Italia S.r.l.) using an increasing linear NaCl concentration. Purified sodin 5 was pooled, dialyzed against water, freeze-dried and stored at −20 °C until use.

During the purification procedure, sodin 5 purity was assessed by SDS-PAGE and RP-HPLC (see Section 2.3), while the enzymatic activity was ascertained by evaluating the ability to depurinate salmon sperm DNA and to release the β-fragment (a hallmark of RIPs) by the rabbit reticulocytes in vitro (see Section 2.4 and Section 2.5).

### 2.3. Analytical Methods

Protein purity was checked by (i) SDS-PAGE (Bio-Rad, Milan, Italy) using a 6.0% stacking and 12% separating polyacrylamide gel with and without reducing agent, and (ii) RP-HPLC using a BioBasic-4 (150 mm × 4.6 mm, 5 µm particle size; Thermo Fisher Scientific, Waltham, MA, USA). For RP-HPLC the following solvents were used: solvent A (Milli-Q water + 0.1% TFA); and solvent B (acetonitrile + 0.1% TFA). Protein elution was carried out using an increasing linear gradient of solvent B, from 5% to 65% of B over 60 min, at 1.0 mL/min, monitoring elution peaks at 214 nm. The protein concentration was achieved by a Pierce BCA Protein Assay Kit (Life Technologies Italia Fil., Monza, Italy).

### 2.4. Polynucleotide:Adenosine Glycosidase Activity

Polynucleotide:adenosine glycosylase activity on salmon sperm DNA (adenine release) was measured according to a previously reported method [37] using salmon sperm DNA (Sigma-Aldrich Solutions, Merk Life Science, Milan, Italy) as a substrate. In addition, this activity was evaluated in the presence of 0.15; 0.30 or 0.60 M NaCl. The highest concentration was chosen considering that NaCl concentration in seawater is generally ~35 g NaCl *per* L (~0.6 M NaCl), while the lowest is the isotonic solution in cells.

### 2.5. rRNA N-Glycosylase Assay

Ribosomal RNA N-glycosylase assay (Endo’s assay) on the lysates of rabbit reticulocytes was conducted as previously described [9,21].

### 2.6. Endonuclease Activity on Supercoiled pUC18 DNA

Endonuclease activity was performed as previously reported [39]. Each reaction contained 3.0 μg protein and 200 ng of pUC18 DNA in 10 mM Tris•acetate, 50 mM NaCl and 50 mM KCl, and a pH of 7.8 with or without 5.0 mM MgCl_2_ (final volume 10 μL). In addition, this activity was evaluated in the presence of 0.15, 0.30, or 0.60 M NaCl. Samples were incubated for 1 h at 37 °C, run on agarose gel (0.8%) in TAE buffer (0.04 M Tris, 0.04 M acetate, 1.0 mM EDTA, and pH 8.0) and visualized by ethidium bromide staining (0.50 µg/mL). Before electrophoretic separation, samples were treated with 0.5% SDS in order to avoid interactions between plasmid and basic protein, which interfere with electrophoretic migration. HindIII linearization was obtained by incubating 250 ng of pUC18 with 1.5 units of HindIII (Amersham Life Sciences Inc., Arlington, MA, USA) according to manufacturer instructions.

### 2.7. Fungal Cultures

Halophilic and halotolerant fungi, previously isolated from Sečovlje salterns (Piran, Slovenia) were selected for the experiment. Deep frozen strains *Aspergillus flavus* (EXF-2368), *Aureobasidium melanogenum* (EXF-9906), *Candida parapsilosis* (EXF-517), *Hortaea werneckii* (EXF-225), *Rhodotorula mucilaginosa* (EXF-10514), and *Trimatostromma salinum* (EXF-295) were recovered from the Culture Collection of Extremophiles (Ex), Infrastructural Center Mycosmo, Biotechnical Faculty, University of Ljubljana, Slovenia. Each fungus was inoculated in triplicates on PDA medium and incubated at 25 °C for 7 days.

To obtain conidia and cells of yeasts and yeast-like fungi, a full loop of *A. melanogenum*, *C. parapsilosis*, *H. werneckii*, and *R. mucilaginosa* were added in triplicates into sterile 150 mL Erlenmayer flasks with 25 mL PDB. Fungal suspensions were incubated at 25 °C for 20 h, shaking at 180 rpm. Conidia of filamentous *A. flavus* and meristematic *T. salinum* were obtained by gently scraping the surface of the cultures with a sterile glass rod after the addition of 5 mL of PDB. Spore suspensions were collected in triplicates into sterile 15 mL Eppendorf flasks.

The concentrations of yeasts and conidia were quantified in a Neubauer chamber and diluted with PDB to reach the concentration of 7.0 × 10^4^ CFU/mL. This concentration was used to test the antifungal activity of sodin 5.

### 2.8. Antifungal Activity

Growth inhibition assays of sodin 5 were performed in 96-well microtiter plates. Conidia or yeast cells of the above-described fungi were suspended in PDB medium with the addition of sodin 5 to reach the final volume per well (200 µL). Conidia or yeast cells were presented in each well in a 1000–1400 CFU, which was confirmed by inoculating the suspension from control wells on PDA. The final concentrations of sodin 5 against each fungus were 0, 10, 20, and 40 µg/mL, respectively. Three independent experiments with triplicate samples were performed. Microtiter plates were incubated at 25 °C. Fungal growth was followed spectrophotometrically with a Multiscan Spectrum plate reader (Thermo Fisher Scientific, New York, NY, USA) for 0, 24, 48, and 72 h. To also follow the effect of sodin 5 into the stationary growth phase, the fungi were monitored for a maximal time of 168 h.

### 2.9. Cytotoxic Activity

For the in vitro cytotoxicity assay, Sf9 cells were plated in sterile 96-well microtiter plates (TPP, Trasadingen, Switzerland) at a cell density of 2.5 × 10^4^ cells per well (100 µL of cell suspension per well). For preparing the cell suspension, ESF 921 medium supplemented with penicillin (100 U/mL) and streptomycin (100 µg/mL; both Gibco, Birmingham, AL, USA) was used. After an initial 3 h incubation, during which cells attached to the bottom of the wells, sodin 5 in antibiotic-supplemented growth medium was added to final concentrations of 1.0, 5.0, 10, 50, 100, 500, and 1000 ng/mL. After 48 h of incubation under cell growth conditions (27 °C, 100% humidity), the cytotoxicity was determined using an MTT (3-(4,5-dimethylthiazol-2-yl)-2,5-diphenyltetrazolium bromide) test, as previously reported [40]. Briefly, the growth medium was removed and attached cells were gently washed with phosphate-buffered saline (PBS) pH 7.4. Next, MTT dissolved in PBS (0.5 mL per mL) was added. After 3 h incubation at 27 °C, the cell supernatant was removed and formed formazan crystals were dissolved in dimethyl sulfoxide (100 µL per well). Absorbance at 570 nm, proportional to the number of viable cells, was measured using microplate reader Sunrise (Tecan, Gröding, Austria). The viability ratio (%) was calculated as the ratio between A_570_ (treated) vs. A_570_ (control). Each protein concentration was tested in three technical repetitions and in three individual experiments.

### 2.10. N-Terminal Amino Acid Sequencing

N-terminal amino acid sequencing of desalted sodin 5 was performed by automated Edman degradation on a PPSQ-53A Gradient System protein sequencer (Shimadzu, Kyoto, Japan).

### 2.11. Mass Spectrometry

A total of 1 µg protein was reduced with 10 mM tris(2-carboxyethyl)phosphine (TCEP) and alkylated with 40 mM chloroacetamide (CAA) in 10 µL 50 mM NH_4_HCO_3_ containing 6.0 M urea in the dark at room temperature for 30 min. The sample was then diluted to 100 µL with 25 mM NH_4_HCO_3_ and hydrolysed with trypsin or chymotrypsin at a protein-to-enzyme ratio of 50:1 overnight at 37 °C. The reaction was stopped by the addition of 1.0 µL trifluoroacetic acid (TFA). The resulting peptides were purified using homemade StageTips C18 and analysed using a Compact ESI-qTOF (Bruker Daltonics, Bremen, Germany) mass spectrometer (MS) coupled to an Acquity UPLC (Waters, Milford, MA, USA). The tryptic peptides were first loaded onto a trap Waters ACQUITY UPLC M-Class Symmetry C18 column and washed with 0.1% (*v*/*v*) formic acid in water at a flow rate of 10 µL/min for 5 min. The bound peptides were then separated on an analytical Bruker Daltonics PepSep C18 column with a gradient of 5% to 45% solvent B (0.1% formic acid in acetonitrile) in 30 min at a flow rate of 0.4 µL/min, followed by a column wash with 90% and 5% solvent B for 5 and 10 min. The electrospray ion source (CaptiveSpray NanoBooster, Bruker Daltonics, Bremen, Germany) operated in positive mode at a source voltage of 1500 V, a drying gas flow of 3.0 L/min and a drying temperature of 150 °C. The MS and tandem mass spectrometry (MS/MS) spectra were acquired in the m/z range of 150-2200 using the appropriate data-dependent acquisition (DDA) method from the oToF Control software v6.3.0.5 (Bruker Daltonics, Bremen, Germany). The collected spectra were exported as Mascot generic files (MGF) and searched by the Mascot software (Matrix Science Ltd., London, UK) (version 2.8) against the sequences of plant ribosome-inactivating proteins extracted from the National Centre for Biotechnology Information non-redundant (NCBInr) database on 30 January 2024. The following parameters were used: 20 ppm peptide and 0.6 Da fragment mass error tolerance, 3 enzyme missed cleavages, 2+ and 3+ peptide charges, carbamidomethyl-Cys as fixed and oxidised Met as variable modification, and an automatic Decoy database search.

### 2.12. Statistical Analysis

Statistical analyses of fungal growth inhibition assays were performed by using GraphPad Prism 8 software (GraphPad Software Inc., San Diego, CA, USA).

## 3. Results and Discussion

### 3.1. Purification of Sodin 5

The purification of sodin 5 was performed according to the procedure reported by Landi et al., 2022 [21]. Briefly, the crude soluble proteins extracted from *S. soda* seeds were clarified by acid precipitation and then subjected to three sequential chromatographic steps: (i) preliminary cation exchange chromatography to select basic proteins; (ii) gel filtration chromatography (30 kDa globular proteins separation); and (iii) cation exchange chromatography.

**Figure 1:**
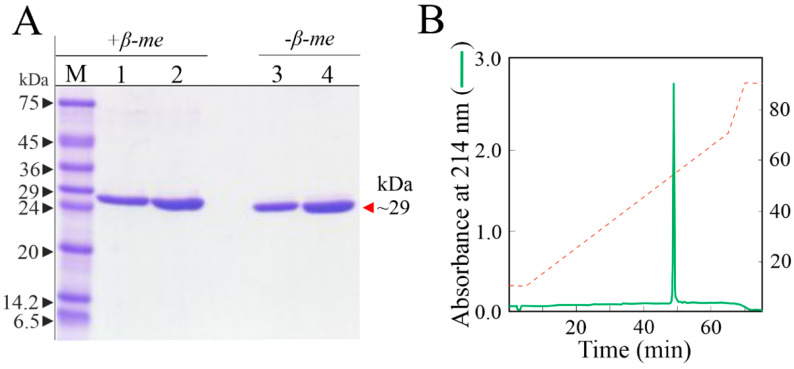
Basic analyses of homogeneity of sodin 5 preparation. (**A**) 12% SDS-PAGE of sodin 5 sample with (+β-me) and without (−β-me) β-mercaptoethanol as reducing agent. Lane M, molecular markers; lanes 1–3 and 2–4, 2.5 and 5.0 µg of sodin 5 samples, respectively. (**B**) RP-HPLC chromatographic profile of sodin 5 preparation (100 µg) by C-4 analytical column.

The purity of sodin 5 for biological and structural studies was assessed by SDS-PAGE with and without reducing agent and RP-HPLC (Figure 1).

### 3.2. Effect of Sodin 5 on the Growth of Halophilic Fung

**Figure 2:**
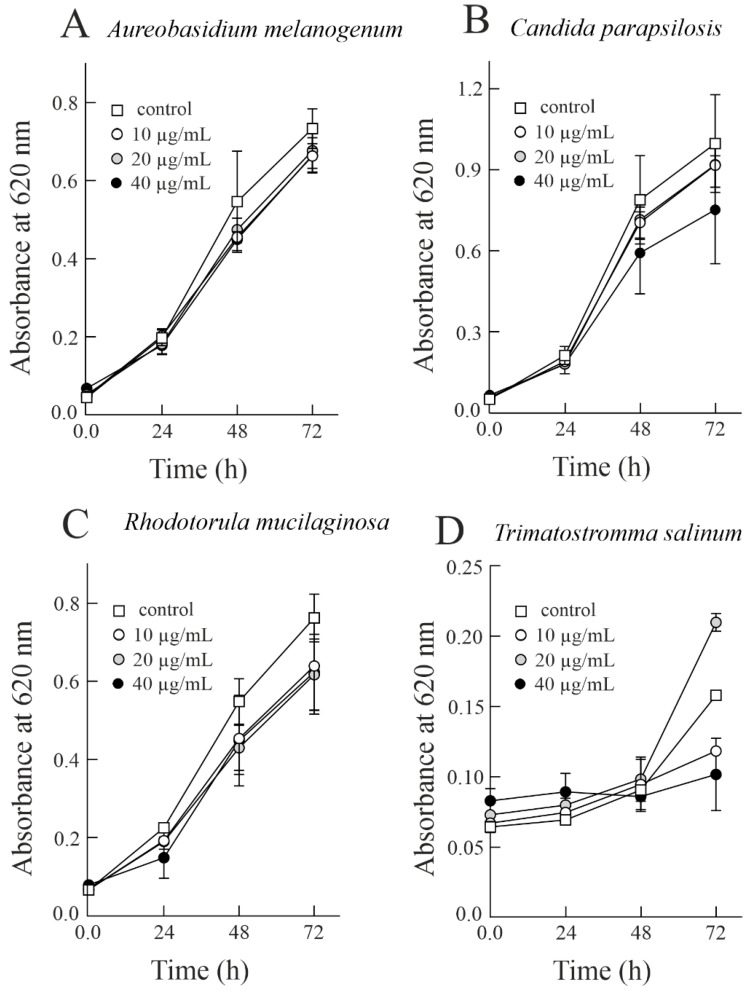
Antifungal activity of sodin 5. The activity of sodin 5 was tested against *Aureobasidium melanogenum* (**A**), *Candida parapsilosis* (**B**), *Rhodotorula mucilaginosa* (**C**), and *Trimmatostromma salinum* (**D**). Fungal conidia were grown at 25 ± 1 °C in PDB medium with the addition of sodin 5 in three different concentrations. Growth was measured by an increase in absorbance at 620 nm.

Considering that *S. soda* has the ability to grow in and colonise hypersaline environments [23], it is reasonable to test the toxicity of sodin 5 against parasitic and competing organisms (e.g., fungi) of *S. soda* able to colonise the same hypersaline ecosystems [41]. In this framework, we verified the antifungal action of this type 1 RIP at three different concentrations (10, 20, and 40 µg/mL; corresponding to 0.34, 0.69, and 1.38 µM, respectively) towards halophilic fungi, *A. melanogenum*, *H. werneckii*, *R. mucilaginosa*, *T. salinum* and *C. parapsilosis* and the halotolerant fungus *A. flavus*. As shown in Figure 2, sodin 5 was able to slow the fungal growth of halophilic fungi in a concentration-dependent manner. In particular, as reported in Table 1, *A. melanogenum*, *C. parapsilos*is, *R. mucilaginosa*, and *T. salinum* treated with the highest concentration of sodin 5 (40 µg/mL; 1.38 µM) resulted in 9.07, 24.38, 18.23, and 35.28% fungal growth inhibition, respectively, after 72 h of growth.

**Figure 3:**
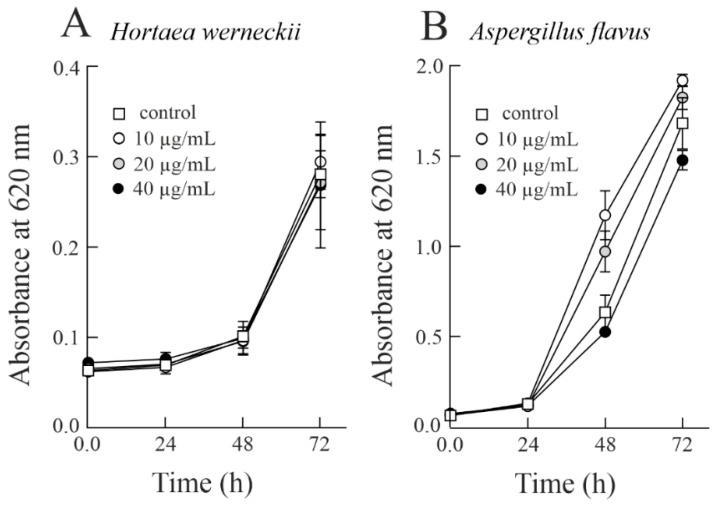
Antifungal activity of sodin 5 against halophilic and halotolerant fungi. Conidia of *Hortaea werneckii* (**A**) and *Aspergillus flavus* (**B**) were grown at 25 ± 1 °C in PDB medium with the addition of sodin 5 in three different concentrations added at 0 h. Fungal growth was measured by an increase in absorbance at 620 nm.

On the other hand, *H. werneckii* was the least sensitive to the treatment with sodin 5, resulting in a very slight inhibition of fungal growth (4.24%) at the highest sodin 5 tested dose (40 µg/mL; 1.38 µM) after 72 h treatment. The same sodin 5 concentration inhibited the growth of halotolerant fungus *A. flavus* by 12.16%; see Figure 3 and Table 1.

**Figure 4:**
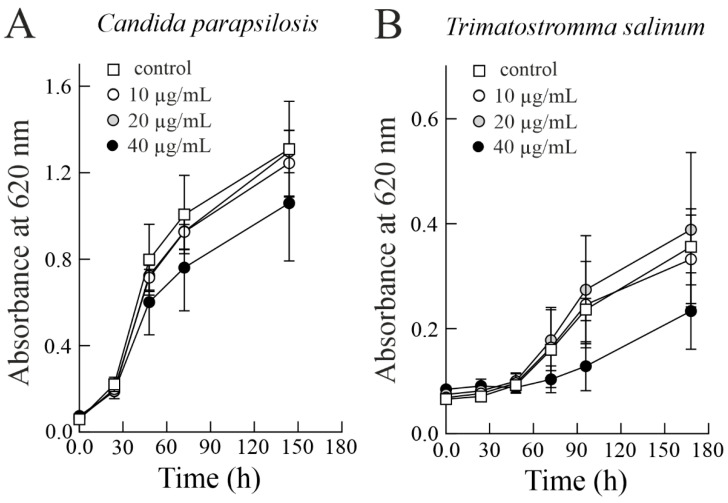
Antifungal activity of sodin 5 against *Candida parapsilosis* (**A**) and *Trimmatostromma salinum* (**B**). Conidia were grown at 25 ± 1 °C in PDB medium with the addition of sodin 5 in three different concentrations added at 0 h. Fungal growth was measured as an increase in absorbance at 620 nm.

Finally, considering that *C. parapsilosis* and *T. salinum* show a higher susceptibility to sodin 5, we decided to extend the incubation time. This is of interest considering both the possible inactivation (i.e., denaturation) of the toxin under the experimental conditions, and the potential reduction in sodin 5 toxicity due to the presence of hydrolytic extracellular enzymes (e.g., peptidases and proteases) released by the fungi under analysis [42,43]. As shown in Figure 4, the concentration-dependent antifungal effect of sodin 5 towards *C. parapsilosis* and *T. salinum* was maintained even after prolongation of the incubation time. Indeed, after ~150 h of incubation, the two fungi treated with the highest concentration of sodin 5 (40 µg/mL; 1.38 µM) resulted in 19.08 (after 144 h) and 34.46% (after 168 h) inhibition of fungal growth, respectively. The inhibition of fungal growth decreased 0.78- and 0.98-fold for *C. parapsilosis* and *T. salinum*, respectively, after these fungi were incubated with sodin 5 for longer times (~150 h).

The same study was also carried out by prolonging the incubation time (~150 h) of sodin 5 with *A. melanogenum*, *H. werneckii*, *R. mucilaginosa,* and *A. flavus* (Figure S1). In this case, the fungal growth inhibition percentages in the presence of the highest concentration of sodin 5 (40 µg/mL) were 4.40, 5.69, 22.11, and 5.65% for *A. melanogenum*, *H. werneckii*, *R. mucilaginosa* and *A. flavus*, respectively. The inhibition of fungal growth increased 1.34- and 1.21-fold when *H. werneckii* and *R. mucilaginosa* were incubated in the presence of the highest concentration of sodin 5 (40 µg/mL) for ~150 h, respectively. Vice versa, the fungal growth inhibition in the same experimental condition decreased 0.48- and 0.46-fold for *A. melanogenum* and *A. flavus*, respectively.

Although all tested fungi have been previously isolated from salterns, and are able to sustain higher concentrations of salt, their primary ecological niches are not necessarily salty habitats. Among the tested fungi, only *Hortaea werneckii* and *Trimmatostroma salinum* are closely related to salterns [44,45,46] and as such likely co-exist with *S. soda*. The growth response of *H. werneckii* to sodin 5 may thus indicate a symbiotic relation with *S. soda*. *Trimmatostroma salinum* is a heavily melanised fungus, with thick cell walls. Responses to the harsh environment include also the formation of meristematic clumps and consequently very slow growth [47]. Meristematic clumps may also affect the measurements because they cannot be fully dissolved to the single-cell level. Thus, the experiment with sodin 5 was also prolonged in order to reach the active growth phase of the fungus. Fungal growth slowed when exposed to the maximal protein concentration, and unlike *H. werneckii,* this may indicate an antagonistic relationship with *S. soda*. *Aspergillus flavus* is considered a ubiquitous fungus and mainly isolated from dust, sand, soil, and plant material [35,48,49], but it was also isolated from seawater and salterns [50,51]. Its specialization in degrading plant material [52] likely provides the ability to sustain and even grow at low concentrations of sodin 5 (10 and 20 µg/mL). *Aureobasidium melanogenum* is associated with freshwater [53], and is not commonly isolated from saline habitats [35]. However, as with *H. werneckii*, *A. flavus* and *T. salinum*, also *A. melanogenum* produce melanin, which is frequently considered an efficient barrier against the defence mechanisms of the host [53,54] and may explain its resistance to sodin 5.

Finally, we tested two ubiquitous yeasts, *Candida parapsilosis* and *Rhodotorula mucilaginosa*, which are often isolated from habitats associated with human and anthropogenic pollution [55,56]. They can be isolated from seawater and salty mud [57,58], but are not primarily associated with salterns or halophytes, which may partly explain their susceptibility to sodin 5. While *C. parapsilosis* growth was inhibited at higher concentrations (20 and 40 µg/mL) of the protein, *R. mucilaginosa* growth was inhibited already at the lowest concentration (10 µg/mL). Among the tested fungi, these yeasts did not produce the excess of melanin [59,60,61,62] and grew faster, which likely enhanced the effect of sodin 5. Apart from *T. salinum*, *R. mucilaginosa* was most affected by the protein. Besides the lack of melanin, one of the reasons might be also the difference in its proliferation cycle [63], since it is the only tested fungus from the kingdom Basidiomycota, while the rest belong to Ascomycota.

In the conditions set in the study, none of the tested fungi reached EC_50_, with the maximal concentration of sodin 5 being 40 µg/mL. To better understand the effect of sodin 5 on halophilic and halotolerant fungi, future studies should focus on a broader concentration range. This would give us an insight into possible hormesis effects, as well as determine effective concentrations that could be used in the biocontrol of *S. soda* crops.

### 3.3. Cytotoxicity of Sodin 5 to Insect Sf9 Cell Line

**Figure 5:**
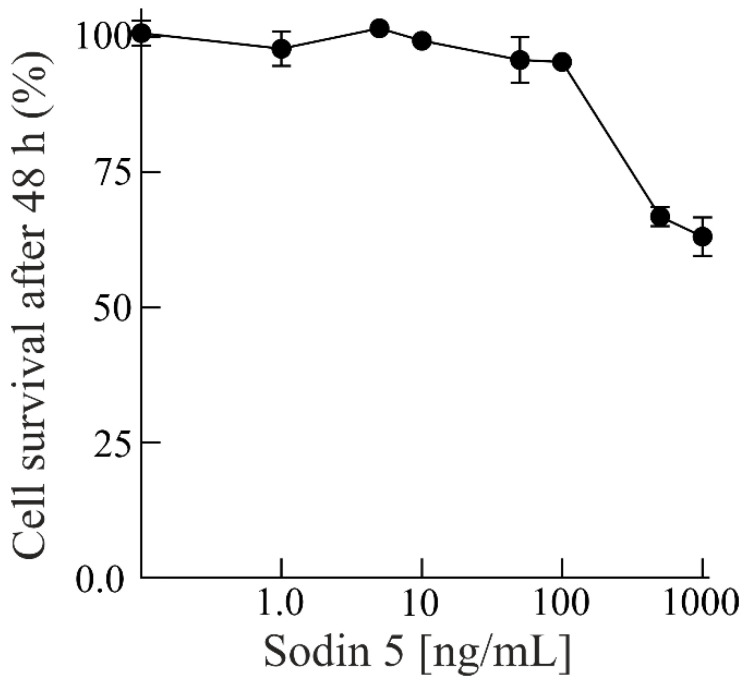
Effect of sodin 5 on survival of insect Sf9 cells. Data are means  ±  SD from three independent experiments.

Recently, after the pioneering work of Bertholdo-Vargas et al. [64], several experimental studies highlighted the insecticidal activity of RIPs in plant defence needed to counter the action of insect attacks [17]. In light of this, the cytotoxic activity of sodin 5 was also assayed on insect Sf9 cells. The results display that sodin 5 is cytotoxic against Sf9 at higher concentrations assayed (500 and 1000 ng/mL) after 48 h of incubation (Figure 5). In particular, sodin 5 decreased the survival of insect cells to 63% at 1000 ng/mL (34.5 nM).

Thus, this finding also highlights the possible involvement of sodin 5 in the defence mechanisms of *S. soda* against insect pests.

Overall, the different effects of sodin 5 most likely depend on its ability to cross both insect and fungal cell membranes, as well as the fungal cell wall. In addition, the cytotoxicity of type 1 RIPs may be due to a different intracellular routing, although not much is known about the pathway(s) followed by type 1 RIPs. Indeed, the absence of the lectin moiety considerably limits the entry of type 1 RIPs into cells and justifies their low level of cytotoxicity compared to type 2 RIPs. With regard to trichosanthin, which has been extensively used to elucidate the routing of type 1 RIPs, it is reported that this toxin can enter cells by receptor-mediated endocytosis, following a Golgi-independent routing pathway [65]. However, recently some authors have reported that this toxin could interact with the low-density lipoprotein receptor-related protein megalin, or enter through clathrin-coated vesicles [16].

### 3.4. PNAG Activity of Sodin 5 in the Presence of NaCl

The high NaCl concentrations of seawater (~35 g NaCl per L or 0.6 M NaCl) could affect the enzymatic activity of sodin 5. In this context, we decided to verify its PNAG and endonuclease activity using salmon sperm DNA and supercoiled pUC18 DNA as substrates, respectively. Indeed, it is known that several RIPs possess endonuclease activity on supercoiled plasmid DNA producing relaxed or linear plasmids, which increases in the presence of divalent ions, such as Mg^2+^ [39]. These properties improve the biological action of RIPs against pathogenic microorganisms or viruses [12,66]. We tested both enzymatic activities of sodin 5 in the absence or presence of increasing NaCl concentration.

**Figure 6:**
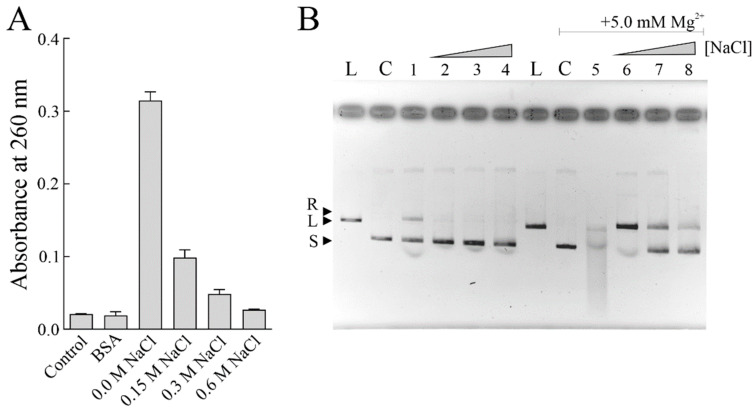
Dependence of enzymatic activities of sodin 5 on NaCl. (**A**) Polynucleotide: adenosine glycosylase activity of BSA (negative control; 3.0 µg) or sodin 5 assayed on salmon sperm DNA. Sodin 5 (3.0 μg) was assayed in the absence or presence of increasing NaCl concentrations as described in Materials and Methods. Data represent the mean of two independent experiments, repeated in triplicate. (**B**) Endonuclease activity of sodin 5 on pUC18 DNA. Samples containing 200 ng/10 µL of plasmid DNA were incubated with 3.0 μg of sodin 5 as described in Materials and Methods. Sodin 5 was incubated in the absence (lane 1) or presence of 0.15, 0.30, and 0.60 M NaCl, lanes 2, 3, and 4, respectively. The same conditions were tested in the presence of Mg^2+^, lanes 5, 6, 7, and 8, respectively. L: pUC18 DNA was previously linearized using EcoRI. R, L, and S indicate relaxed, linear, and supercoiled forms of pUC18, respectively; 0.8% agarose gel electrophoresis.

As reported in Figure 6A, the PNAG activity of sodin 5 decreased in the presence of increasing NaCl concentrations. The same behaviour was observed also when the endonuclease activity of sodin 5 was evaluated in the absence (lane 1, Figure 6B) or the presence of increasing NaCl concentrations (lanes 2, 3, and 4, Figure 6B). On the other hand, by adding Mg^2+^ ions, the endonuclease activity, the highest in the absence of NaCl (lane 5, Figure 6B), was reduced by increasing the NaCl concentration (lanes 6, 7, and 8, Figure 6B).

Overall, these results suggest that although sodin 5 exhibits antifungal activity against halophilic fungi, its enzymatic activity is affected by NaCl.

A possible explanation for the lower enzymatic activity of sodin 5 in the presence of NaCl could be due to the structural changes in the DNA substrate [67]. It is known that monovalent ions such as Na^+^ stabilize DNA molecules interacting with phosphates and the grooves, altering the intramolecular fluctuations, which could be useful for enzyme-substrate interactions [68].

### 3.5. The Partial Amino Acid Sequence of Sodin 5

To gain insight into the primary structure of sodin 5, the native protein was first desalted by RP-HPLC and then subjected to N-terminal amino acid sequencing. No sequence was obtained even when 100 pmol of the protein was analysed, indicating that the N-terminal amino acid residue of sodin 5 was blocked.

To obtain structural information on sodin 5, the S-carbamidomethylated protein was fragmented with trypsin or chymotrypsin and the resulting peptides were analysed by high-resolution nano-LC-tandem mass spectrometry. The obtained spectral data were searched against an internal database containing plant RIP sequences reported in the NCBInr database. We identified sodin 5 as a type 1 RIP as it shares sequence identity with antiviral RIP (AEY75258.1) from four-wing saltbush (*Atriplex canescens*; Amaranthaceae family) and the RIPs (BAB83507.1, BAH56516.1) from spinach (*Spinacia oleracea*; Amaranthaceae family) (Table 2 and Figure S2). Peptides T-5 and C-1 of sodin 5 overlap with the catalytic site of RIPs, as they include Glu157 and Arg160 (the numbering refers to the amino acid sequence of the AEY75258.1), the amino acid residues necessary for the enzymatic activity of RIPs [69,70,71,72]. A UniProt database search revealed additional RIPs with parts homologous to the amino acid sequence of sodin 5. Sequences of these, better-characterized RIPs, are included in the sequence alignment in Figure S2.

Overall, the data obtained allowed us to confirm that sodin 5 is homologous to type 1 RIPs. We identified the sequence of 57 amino acid residues in sodin 5, including amino acid residues characteristic of the catalytic site of RIPs [71]. Considering that type 1 RIPs typically consist of ~250 amino acid residues, we have revealed ~23% of the primary structure of sodin 5.

## 4. Conclusions

The cultivation of plants like *S. soda* in saline soils is becoming economically interesting due to the increasing changes in the human population and the lack of cultivable crops. Understanding their defence mechanisms against microbes and animals could significantly enhance their production in arid and salty areas. One of the mechanisms in *S. soda* is the production of RIP protein sodin 5. Several studies highlighted that RIPs are expressed in plants as defence molecules against pathogens and insect pest attacks. To improve our knowledge of sodin 5, we have collected substantial structural information on this protein and confirmed that it belongs to the type 1 RIPs of the Amaranthaceae family. Considering the potential use of sodin 5 as a biopesticide in salty habitats, we determined the dependence of its enzymatic activity on NaCl concentrations.

Additionally, the antifungal activity of sodin 5 was demonstrated on halophilic and halotolerant fungi, originating either from salterns or being introduced into seawater due to anthropogenic pollution. Sodin 5 exhibited the highest antifungal activity against *Candida* and *Rhodotorula*, yeasts of anthropogenic origin, and against meristematic, slow-growing *T. salinum*. It was less effective against halophilic species of melanised fungi *H. werneckii*, *A. melanogenum*, and *A. flavus*.

Together with the cytotoxic effect of sodin 5 on the insect cell line, this opens the perspective to test higher concentrations of sodin 5 on phytopathogenic fungi and other animal cell lines in order to use this protein as a biocontrol agent not only against endemic fungi but also on those introduced into seawater and shore by anthropogenic pollution.

## Figures and Tables

**Table 1:** Inhibition of fungal growth in the presence of sodin 5. Growth inhibition in percentage after 72 h of halophilic and halotolerant fungi incubated with three different concentrations of sodin 5.

Fungal Strain	EXF-No ^1^	Growth Inhibition (%)		
		10 µg/mL	20 µg/mL	40 µg/mL
*Aspergillus flavus*	EXF-2368	−14.12	−8.48	12.16
*Aureobasidium melanogenum*	EXF-9906	9.29	7.52	9.07
*Candida parapsilosis*	EXF-517	7.98	7.82	24.38
*Hortaea werneckii*	EXF-225	−4.82	3.29	4.24
*Rhodotorula mucilaginosa*	EXF-10514	16.17	19.06	18.23
*Trimatostromma salinum*	EXF-295	−2.71	−11.48	35.28

^1^, number designated to fungal strain in the Culture Collection of Extremophiles (Ex), Infrastructural Center Mycosmo, Biotechnical Faculty, University of Ljubljana, Slovenia.

**Table 2:** Sodin 5 peptides identified by mass spectrometry. Mass spectra were generated using the Compact ESI-qTOF mass spectrometer and interpreted by searching against the plant RIP sequences from the NCBInr database.

Peptide	Peptide Sequence	Number of Identified +2H Spectra	Number of Identified +3H Spectra	Calculated +1H Peptide Mass (Da)	Originating Protein [Species]	Accession Number	Molecular Mass (Da)
Trypsin cleavage							
T-1	NDLYVVAFADK	2	0	1254.64	antiviral ribosome-inactivating protein [*Atriplex canescens*]	AEY75258.1	30849.80
T-2	LSFPLGFDNLK	8	0	1250.68	antiviral ribosome-inactivating protein [*Atriplex canescens*]	AEY75258.1	30849.80
T-1	ILSLENNWGAISK	1	0	1444.78	antiviral ribosome-inactivating protein [*Atriplex canescens*]	AEY75258.1	30849.80
T-4	NDMGLLK	44	0	790.41	antiviral ribosome-inactivating protein [*Atriplex canescens*]	AEY75258.1	30849.80
T-5	FFLIAIQMVAEAAR	23	21	1595.86	ribosome-inactivating protein [*Spinacia oleracea*]	BAB83507.1, BAH56516.1	35769.30
Chymotrypsin cleavage							
C-1	LIAIQMVAEAARF	5	0	1448.79	antiviral ribosome-inactivating protein [*Atriplex canescens*] ribosome-inactivating protein [*Spinacia oleracea*]	AEY75258.1 BAB83507.1, BAH56516.1	30849.80 35769.30

## Data Availability

The data presented in this study are available on request from the corresponding author.

## Supplementary Materials

The following supporting information can be downloaded at: https://www.mdpi.com/article/10.3390/biom14030336/s1. Figure S1. Antifungal activity of sodin 5 against *Aureobasidium melanogenum* (A), *Rhodotorula mucilaginosa* (B), *Hortaea werneckii* (C), and *Aspergillus flavus* (D) after ~150 h of incubation. Figure S2. The amino acid sequence alignment of the RIPs showing homology with sodin 5.
