# Supplementary material for: Biocontrol Potential of Sodin 5, Type 1 Ribosome-Inactivating Protein from Salsola soda L. Seeds
Source: Biomolecules. 2024 Mar 12;14(3):336. doi: 10.3390/biom14030336 (PMC10967906; doi:10.3390/biom14030336)
Supplement: Supplementary file 1 [file biomolecules-14-00336-s001.zip › biomolecules-2903698-supplementary.pdf]

Supplementary Materials

Biocontrol potential of sodin 5, type 1 ribosome-inactivating protein from *Salsola soda* L. seeds

Monika Novak Babič [1, ‡], Sara Ragucci [2, ‡], Adrijana Leonardi [3], Miha Pavšič [4], Nicola Landi [2, 5], Igor Križaj [3], Nina Gunde-Cimerman [1], Kristina Sepčić [1] and Antimo Di Maro [2, *]

[1] Department of Biology, Biotechnical Faculty, University of Ljubljana, 1000-Ljubljana, Slovenia

[2] Department of Environmental, Biological and Pharmaceutical Sciences and Technologies (DiSTABiF), University of Campania 'Luigi Vanvitelli', 81100-Caserta, Italy

[3] Department of Molecular and Biomedical Sciences, Jožef Stefan Institute, 1000-Ljubljana, Slovenia

[4] Department of Chemistry and Biochemistry, Faculty of Chemistry and Chemical Technology, University of Ljubljana, Ljubljana, Slovenia

[5] Institute of Crystallography, National Research Council of Italy, 81100-Caserta, Italy

‡ The first two authors contributed equally to this study

* Correspondence: antimo.dimaro@unicampania.it (A.D.M.)

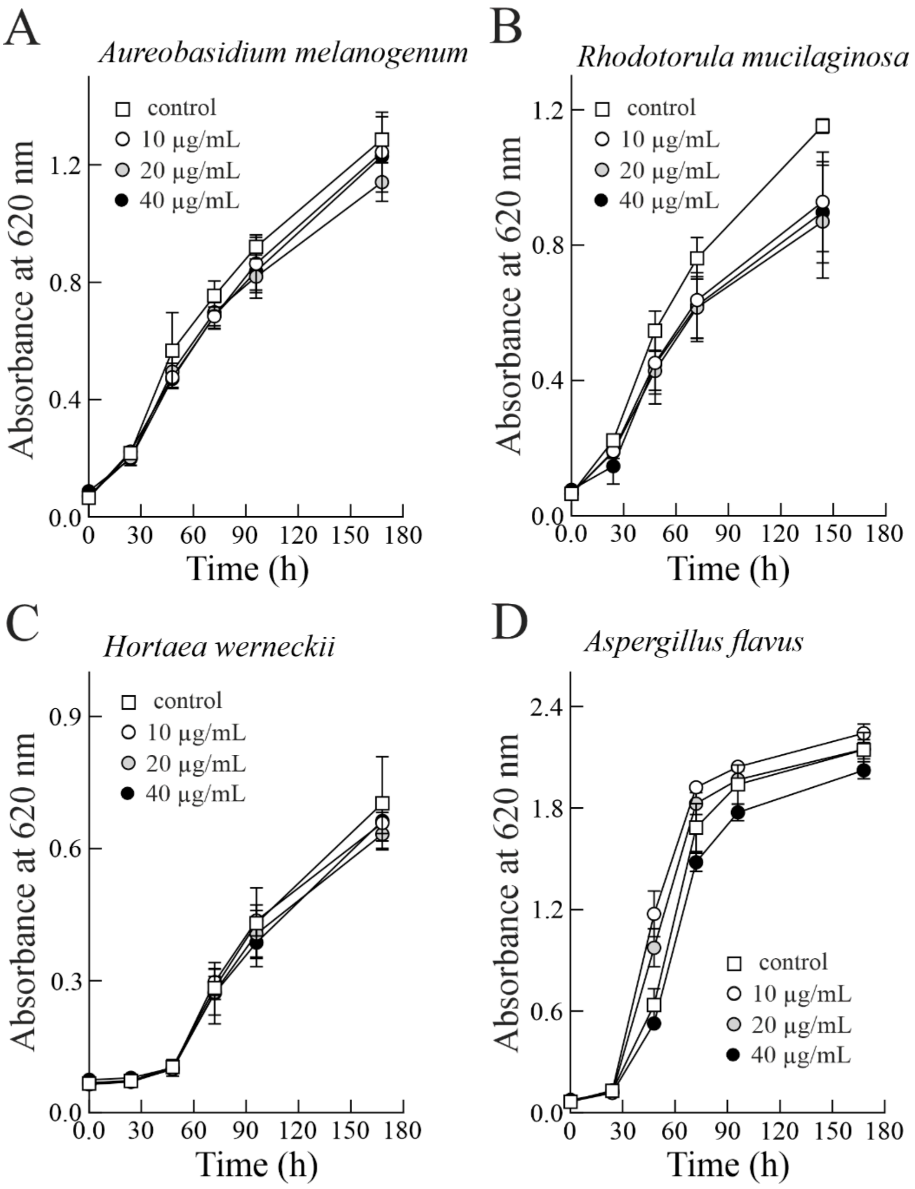

Figure S1. Antifungal activity of sodin 5 against *Aureobasidium melanogenum* (A), *Rhodotorula mucilaginosa* (B), *Hortaea werneckii* (C) and *Aspergillus flavus* (D) after ~150 h of incubation. Fungal conidia were grown at 25±1 °C in PDB medium with the addition of sodin 5 in three different concentrations. Fungal growth was measured as an increase in absorbance at 620 nm.

```
AEY75258.1            1  -----------------------QAIRNQVKDPNLVYEGIPMIRAPTKPDTYLLVELESV 37
RIPS_PHYAM            1  ---INTITFDAGNATINKYATFMESLRNEAKDPSLKCYGIPMLPNTNSTIKYLLVKLQ-- 55
RIP2_PHYDI            1  ---VSTITFDVGSATISKYTTFLESLRNQAKDPSLKCYGIPMLPNTNPNPKYLLVELNAK 57
RIPA_PHYAM            1  ---INTITFDVGNATINKYATFMKSIHNQAKDPTLKCYGIPMLPNTNLTPKYLLVTLQ-- 55
RIPP_MIRJA            1  APTLETIASLDLN-NPTTYLSFITNIRTKVADKTEQCTIQKISKTFT--QRYSYIDLI-- 55
BAB83507.1            1  ---YTTVTFELTN-GVTGYSSFMTRLRNAVEAPTRACGLQSTRSTPLPGAEYIHVDLK-- 54
Clustal Consensus     1                           ::. .   .                 *  : *     8

AEY75258.1           38  KN-NKKISLRLALSRNDLYVVAFADKYG--GKVRGHFFPHLNIDTIEKA--KKVFP--DV 90
RIPS_PHYAM           56  --GASLKTITLMLRRNNLYVMGYSDPYD--NKCRYHIFNDIKG-TEYSDVENTLCPSSNP 110
RIP2_PHYDI           58  LSSGEVKSITLMLRRHNLYVMGYSDPYD--NKCRYHIFKDISSTTERKDVETTLCPNASS 115
RIPA_PHYAM           56  --DSSLKTITLMLKRNNLYVMGYADTYN--GKCRYHIFKDISNTTERNDVMTTLCPNPSS 111
RIPP_MIRJA           56  --VSSTQKITLAIDMADLYVLGYSDIANNKG--RAFFFKDVTE-----AVANNFFPGATG 106
BAB83507.1           55  --ISNTQWVTLGIDVKDFYVWGYQDNVRYNGNYRASFLNDAPQ-----AAKNNLFRGSTI 107
Clustal Consensus     9      .   : * :   ::** .: *     .  *  :: .           ...        27

AEY75258.1           91  VEVVQIKYGESYAQIESNAGT-NRLSFPLGFDNLKANMEKVYGMDTSAKDYSKTEARSLL 149
RIPS_PHYAM          111  RVAKPINYNGLYPTLEKKAGVTSRNEVQLGIQILSSDIGKISGQGSFT---EKIEAKFLL 167
RIP2_PHYDI          116  RVSKNISYDSSYPALENKAGR-SRSQVQLGIQILNSDIGKISGVKSFT---DKTEAEFLL 171
RIPA_PHYAM          112  RVGKNINYDSSYPALEKKVGR-PRSQVQLGIQILNSGIGKIYGVDSFT---EKTEAEFLL 167
RIPP_MIRJA          107  TNRIKLTFTGSYGDLEKNGGL-RK-DNPLGIFRLENSIVNIYGKAGD----VKKQAKFFL 160
BAB83507.1          108  ---RTTRFGGNYNSLEPAAGI-TRRNLVLGIQNLDGAIRTVYGKQESELNKGNAEARFFL 163
Clustal Consensus    28         :   *  :*   *   : .  **:  *.  : .: *         : :*. :*  49

AEY75258.1          150  IAIQMVAEAARFKYIQGRAVVTTP---PNDYKILSLENNWGAISKGIRNAVKKVI----- 201
RIPS_PHYAM          168  VAIQMVSEAARFKYIENQVKTNFNRDFSPNDKVLDLEENWGKISTAIHNSKNGAL----- 222
RIP2_PHYDI          172  VAIQMVSEATRFKYIENQVKTNFNRAFYPNAKVLNLEETWGKISMAIHGAKNGAF----- 226
RIPA_PHYAM          168  VAIQMVSEAARFKYIENQVKTNFNRAFYPNAKVLNLEESWGKISTAIHNAKNGAL----- 222
RIPP_MIRJA          161  LAIQMVSEAARFKYISDKIPSEKYEEVTVDEYMTALENNWAKLSTAVYNSKPSTTTATKC 220
BAB83507.1          164  IAIQMVAEAARFKFMEEAIVRNDN-TPDFKRKMVAFQNDWDPISTAIHRAEAAT---PKC 219
Clustal Consensus    50  :*****:**:***::.             .  :  ::: *  :* .:  :   .        77

AEY75258.1          202  ---NPPITLQYPNGATWTVTQVGDIKNDMGLLKYVS----------------------   234
RIPS_PHYAM          223  ---PKPLELKNADGTKWIVLRVDEIKPDVGLLNYVNGTCQAT----------------   261
RIP2_PHYDI          227  ---SKPLELQNADGSKWIVLRVDEIKPDVALLKYVSGSCQAT----------------   265
RIPA_PHYAM          223  ---TSPLELKNANGSKWIVLRVDDIEPDVGLLKYVNGTCQATYQSAMFPHL-------   270
RIPP_MIRJA          221  QLATSPVTISP-W----IFKTVEEIKLVMGLLKSS-----------------------   250
BAB83507.1          220  TRITPTLVISN-TEYRQEVNTVAEIKDDMGLLKYRSTTLSSIASSILDDDEFLESALI   276
Clustal Consensus    78         : :.        .  * :*:  :.**:                          90
```

Figure S2. The amino acid sequence alignment of the RIPs showing homology with sodin 5. AEY75258.1 is an antiviral RIP from four-wing saltbush (*Atriplex canescens*), RIPS_PHYAM and RIPA_PHYAM are the RIPs from the American pokeweed (*Phytolacca Americana*). RIP2_PHYDI is the RIP from ombú (*Phytolacca dioica*), RIPP_MIRJA from the marvel of Peru (*Mirabilis jalapa*) plant and BAB83507.1 the RIP from spinach (*Spinacia oleracea*). S-carbamidomethylated sodin 5 was fragmented with trypsin and, alternatively, with chymotrypsin. Sequences of the resulting peptides were identified using mass spectrometry (see Table 2). In the figure, sequences of the sodin 5 tryptic peptides are marked yellow while sequences of its chymotryptic peptides are underlined. Glu and Arg, constituting the catalytic site of RIPs, are typed red. In Clustal Consensus line, the positions with an identical amino acid residue in all aligned sequences are indicated with an asterisk (*), the

positions, where very similar amino acid residues are found in the aligned sequences (conservative substitutions) are indicated with a colon (:), and the positions, where to some extent similar amino acid residues are found in the aligned sequences (semi-conservative substitutions) are indicated with a dot (.).
